# Dental Anomalies: An Identification Marker in Forensics

**DOI:** 10.7759/cureus.59922

**Published:** 2024-05-08

**Authors:** Shanmathy Sureshbabu, Ramya Ramadoss, Abirami Arthanari, Karthikeyan Ramalingam

**Affiliations:** 1 Department of Forensic Odontology, Saveetha Dental College and Hospitals, Saveetha Institute of Medical and Technical Sciences, Saveetha University, Chennai, IND; 2 Department of Oral Pathology and Oral Biology, Saveetha Dental College and Hospitals, Saveetha Institute of Medical and Technical Sciences, Saveetha University, Chennai, IND; 3 Department of Oral Pathology and Microbiology, Saveetha Dental College and Hospitals, Saveetha Institute of Medical and Technical Sciences, Saveetha University, Chennai, IND

**Keywords:** dental anomalies, forensic identification, human identification, permanent teeth, cusp of carabelli (cc), dental non-morphic traits

## Abstract

Aim

This study aims to evaluate the non-morphological traits of the South Indian population.

Introduction

Dental morphological traits, also known as non-metric dental traits, exhibit variation in appearance both within and between groups. The study analyzed the non-metric traits among the South Indian population, as few variants can be grouped within the population.

Materials and methods

A total of 500 extracted tooth samples were collected. The dental non-metric traits that were evaluated are the cusp of Carabelli (CC), Talon's cusp (TC), shoveled incisor (SI), peg-shaped lateral incisor (PL), protostylid (PR), *Dryopithecus* pattern groove (DP), hypoconulid (HY), parastyle (PA), multiple parastyle (MPA), paracone (PC), Bushman's canine (BC), interruption groove (IG), tuberculum dentale (TD), tuberculum intermedium (TI), radix entomolaris (RE), fusion (F), radiculous premolar (RP), dilaceration (D), dens evaginatus (DE), and enamel pearl (EP).

Results

Out of 20 dental non-metric traits that were evaluated in the study, 14 traits were identified to be common within the population. The prevalence were as follows: cusp of Carabelli (52%), shoveled incisor (8.2%), peg-shaped lateral incisor (7.4%), parastyle (0.8%), multiple parastyle (0.2%), Bushman's canine (0.4%), interruption groove (2.2%), tuberculum intermedium (0.6%), radix entomolaris (39.6%), fusion (2.8%), radiculous premolar (0.2%), dilaceration (58.2%), dens evaginatus (1.2%), and enamel pearl (0.8%) among the South Indian population.

Conclusion

The current study was discovered to have more Carabelli traits, shoveled incisors, radix entomolaris, and dilaceration than other non-metric features. This shows that these characteristics are more prevalent in the South Indian population, which could be one of the strategies used to validate human identification in a forensic context.

## Introduction

In the past, to identify a person's sex, origin, and identity, the morphology of the teeth was studied. Non-metric dental traits are traits that can be passed down genetically but exhibit diversity in expression both within and between populations [1]. Inadequate historical records about previous armed conflicts, natural or mass disasters that produced such human remains, severe damage or fragmentation, missing elements, and poor preservation all make the process of forensic anthropological identification of commingled human remains recovered from diverse contexts more difficult and complex [2]. An essential part of biological anthropology and forensic anthropology, non-metric dental features provide unique insights into the evolutionary history and genetic makeup of the human population. Non-metric attributes, as opposed to traditional metrics, concentrate on the qualitative characteristics of dental morphology, such as accessory cusps, shovel-shaped incisors, and cusp patterns [3]. These features are significant because they are frequent in a specific ethnicity and appear as a pattern in a particular demographic. The occurrence and degree of expression of tooth variants have been shown in numerous studies in dental anthropology to have a coherent congenital relevance and may be indicative of ethnicity, providing crucial information for phylogenetic and/or genetic investigations [4]. Within a certain group, several non-metric dental features are consistent and may be a good indicator of their ancestry. Due to the large mineralized content of the tooth, which makes it steadfast, these non-metric dental qualities have the benefit that their morphological characteristics stay steady unless disturbed by extrinsic factors (particularly dental caries, wasting diseases, trauma, and aesthetic treatments). Therefore, these can be effectively employed in racial identification, which is useful in identifying the person through the antemortem dental record [5].

The mesiopalatal line angle of the maxillary first molar has a little extra cusp called the cusp of Carabelli. Usually seen on the upper first molar, this additional cusp progressively disappears, most likely in the second and third molars. Some people have a complete absence of this cusp, while others have it in different forms [6]. The lingual surfaces of incisors with a shovel-like form have ridges at the lingual margins. In older research, these characteristics were utilized to distinguish between the Caucasian and Mongoloid people. Few dental features have been examined in worldwide research on dental casts, direct clinical evaluation, radiography, and digital photography. Currently, over 135 dental features have been found in the human dental system. Since non-metric qualities' characteristics are simple to see and document, they give us knowledge about genetic and ethnic variations that happen, enabling us to arrange populations following the process of group-specific evolution. However, it should be mentioned that tooth wear and caries might cause some dental features to disappear [7].

Few studies have been done to fully understand the universality of all the traits in an ethnic population, even though dental non-morphic trait studies were carried out in the past [8]. These characteristics are useful markers for identifying genetic lineages and comprehending population dynamics since they are mainly heritable and resistant to environmental factors. As far as we are aware, this is the first study to thoroughly examine the South Indian population. Since the majority of current information on dental anatomy comes from European and American studies [9], indigenous studies are urgently needed because their findings may not apply to the South Indian ethnic population, which has its roots in the Dravidian age. By offering researchers a forensic instrument and a window into the complex web of human variation and lineage, the study of non-metric dental features makes a substantial contribution to understanding the subtle interplay between genetics, adaption, and evolution [10].

## Materials and methods

Study setting

An observational study was conducted with a total of 500 extracted tooth samples that were collected from the tooth repository of Saveetha Dental College and Hospitals. Cochran's formula was used to determine the sample size. A descriptive analysis was performed for the present study.

Statistical analysis

The data was entered into Microsoft Excel, and descriptive analysis was conducted using IBM SPSS Statistics for Windows, Version 17.0 (Released 2008; IBM Corp., Armonk, New York, United States).

Inclusion and exclusion criteria

The extracted teeth were selected based on a few requirements such as non-carious, non-fractured, non-attrited, and non-eroded. Teeth with restorations and dental crowns were also excluded from the study. Twenty non-metric dental traits were considered which is illustrated in Table 1. 

**Table 1:** Characteristics of non-metric dental traits. References: [11-13]

Traits	Features
Cusp of Carabelli	An accessory cusp that varies in size and shape, composed of enamel and dentin with or without pulpal extension in the maxillary molars
Talon's cusp	An extra cusp or cusp-like projection in the palatal surface of the maxillary anterior teeth
Shoveled incisors	Teeth with prominent marginal ridges that surround a deep lingual fossa
Peg-shaped lateral incisor	Lateral incisors that are often pointed resembling a cone
Protostylid	An accessory cusp that is present on the buccal surface of lower molars
*Dryopithecus* pattern groove	The mesiolingual and distobuccal cusps are joined across the ﬂoor of the central fossa to form a five-cusped pattern
Hypoconulid	Posterior-most cusp on the mandibular first molar
Paracone	An accessory cusp on the buccal aspect of the maxillary first molar
Parastyle	A paramolar cusp that appears on the buccal surface of the maxillary molars
Multiple parastyle	Multiple paramolar cusps that appear on the buccal surface of the maxillary molars
Bushman's canine	A mesial ridge on the lingual surface of the canine
Interruption groove	A groove on the upper incisor that meets the cingulum and may continue to the root
Tuberculum dentale	Cingular prominence of maxillary anterior, often seen as a projection of the cingulum
Radix entomolaris	Presence of one or more additional roots in the mandibular molars
Fusion	Union of two separate tooth germs
Radiculous premolar	Presence of one or more additional roots in the premolars
Dilaceration	Change in the axial inclination between the crown and root of the tooth is commonly seen in molars
Dens evaginatus	The outer surface of the tooth forms a bump, often seen in mandibular premolars
Enamel pearl	The globule of enamel formation on the root surface
Tuberculum intermedium	An accessory cusp is present on the buccal cusp of the mandibular molars

To rule out bias, two observers examined the samples, and for maintenance of record, photographs were taken if any trait was observed (kappa value=100%). The frequency of the trait was also analyzed in Table 2.

**Table 2:** Frequency of the trait. UI: upper incisor; LI: lower incisor; UC: upper canine; LC: lower canine; UP: upper premolar; LP: lower premolar; UM: upper molar; LM: lower molar

Traits observed	Teeth evaluated
Cusp of Carabelli (CC)	UM 1
Talon's cusp (TC)	UC
Shoveled incisor (SI)	UI 1, UI 2, LI 1, LI 2
Peg-shaped lateral incisor (PL)	UI 2
Protostylid (PR)	UI 1, UI 2, LI 1, LI 2
*Dryopithecus* groove pattern (DP)	LM 2
Hypoconulid (H)	LM 1
Parastyle (PA)	UM 1, 2, 3
Multiple parastyle (MPA)	UM 1, 2, 3
Paracone (PC)	UM 1, 2, 3
Bushman's canine (BC)	UC
Interruption groove (IG)	UI 1, UI 2
Tuberculum dentale (TD)	UI 1, UI 2, UC
Radix entomolaris (RE)	LM 1, 2, 3
Fusion (F)	NA
Tuberculum intermedium (TI)	LM 1
Radiculous premolar (RP)	UP 1
Dilaceration (D)	UP 1, 2, UM 1, 2, 3, LP 1, 2, LM 1, 2, 3
Dens evaginatus (DE)	LP 1, 2
Enamel pearl (EP)	UM 1, 2, 3

## Results

Out of the 20 defined non-morphic dental traits that were assessed in the present study, 14 were found common in the South Indian population. In the present study, 500 teeth were examined. On the examination, the traits observed were cusp of Carabelli (52%), shoveled incisor (8.2%), peg-shaped lateral incisor (7.4%), parastyle (0.8%), multiple parastyle (0.2%), Bushman's canine (0.4%), interruption groove (2.2%), tuberculum intermedium (0.6%), radix entomolaris (39.6%), fusion (2.8%), radiculous premolar (0.2%), dilaceration (58.2%), dens evaginatus (1.2%), and enamel pearl (0.8%).

In our study, images showing the cusp of Carabelli, interruption groove, peg lateral, parastyle, radiculous premolar, dens evaginatus, enamel pearl, radix entomolaris, Bushman's canine, multiple parastyle, tuberculum intermedium, fusion, and dilaceration are shown in Figures 1, 2, 3, 4, 5, 6, 7, 8, 9, 10, 11, 12, 13.

**Figure 1:**
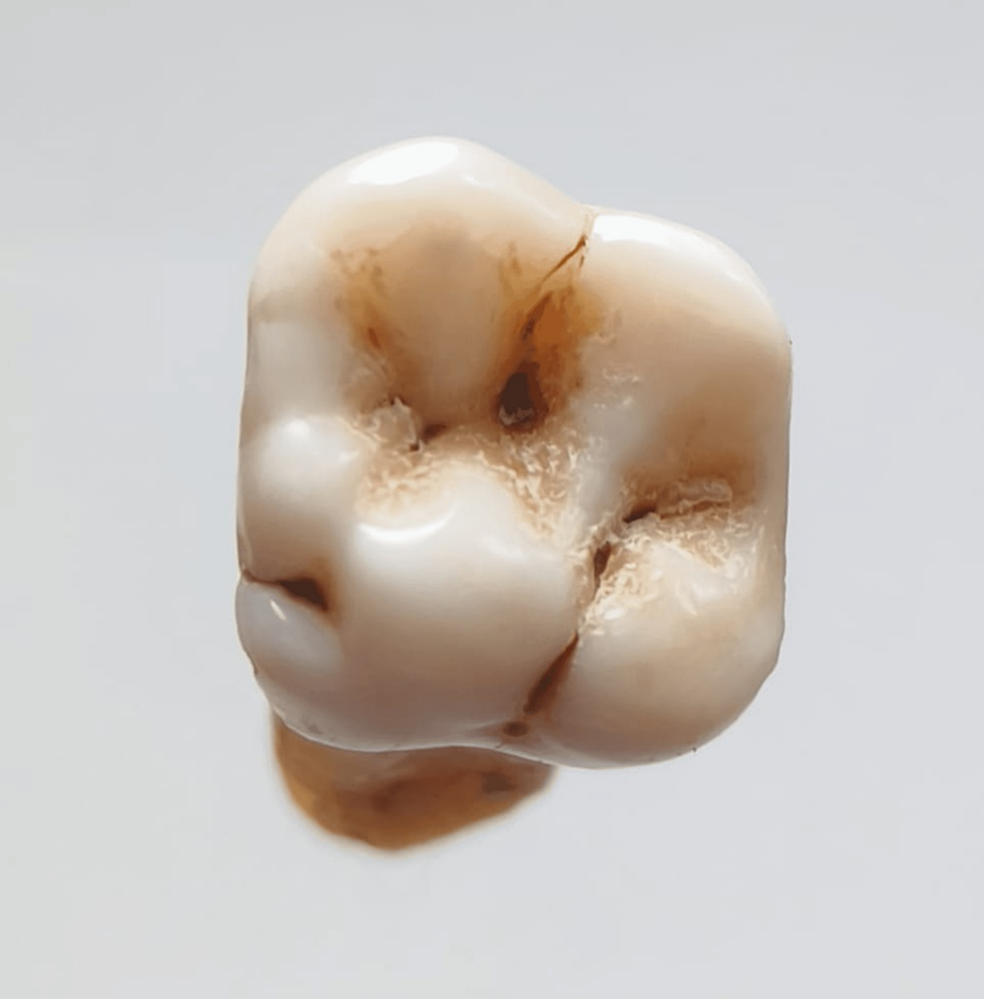
Image showing cusp of Carabelli.

**Figure 2:**
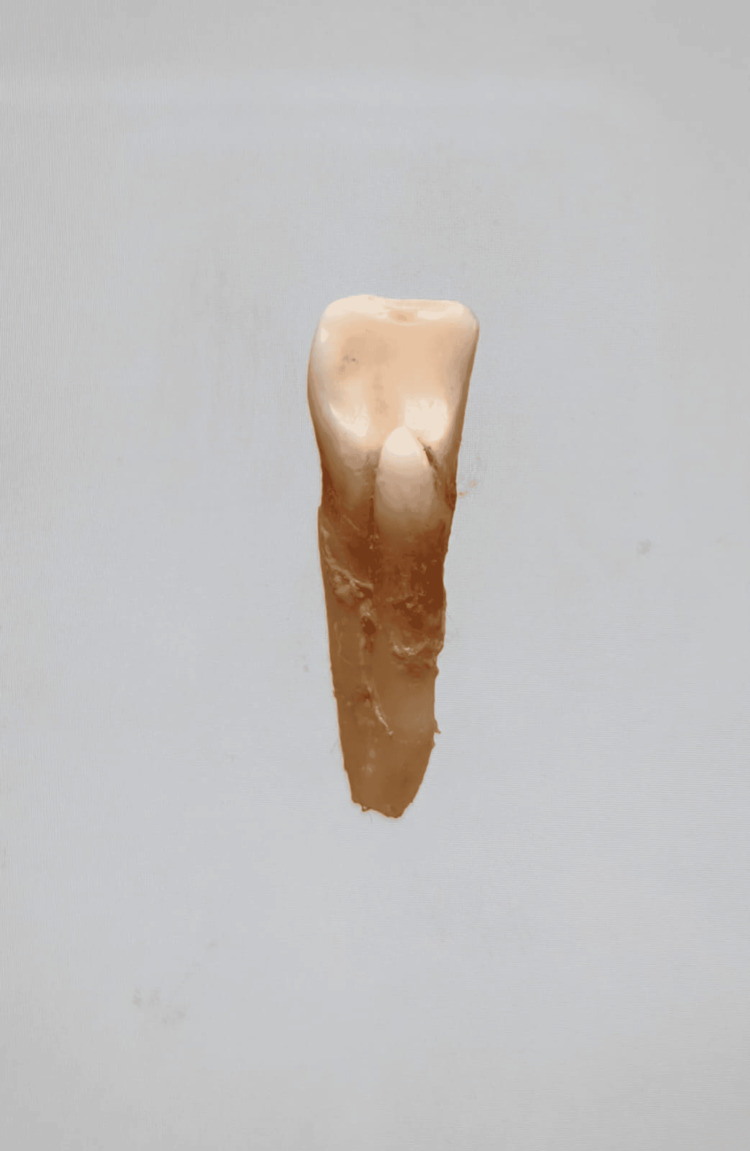
Image showing interruption groove.

**Figure 3:**
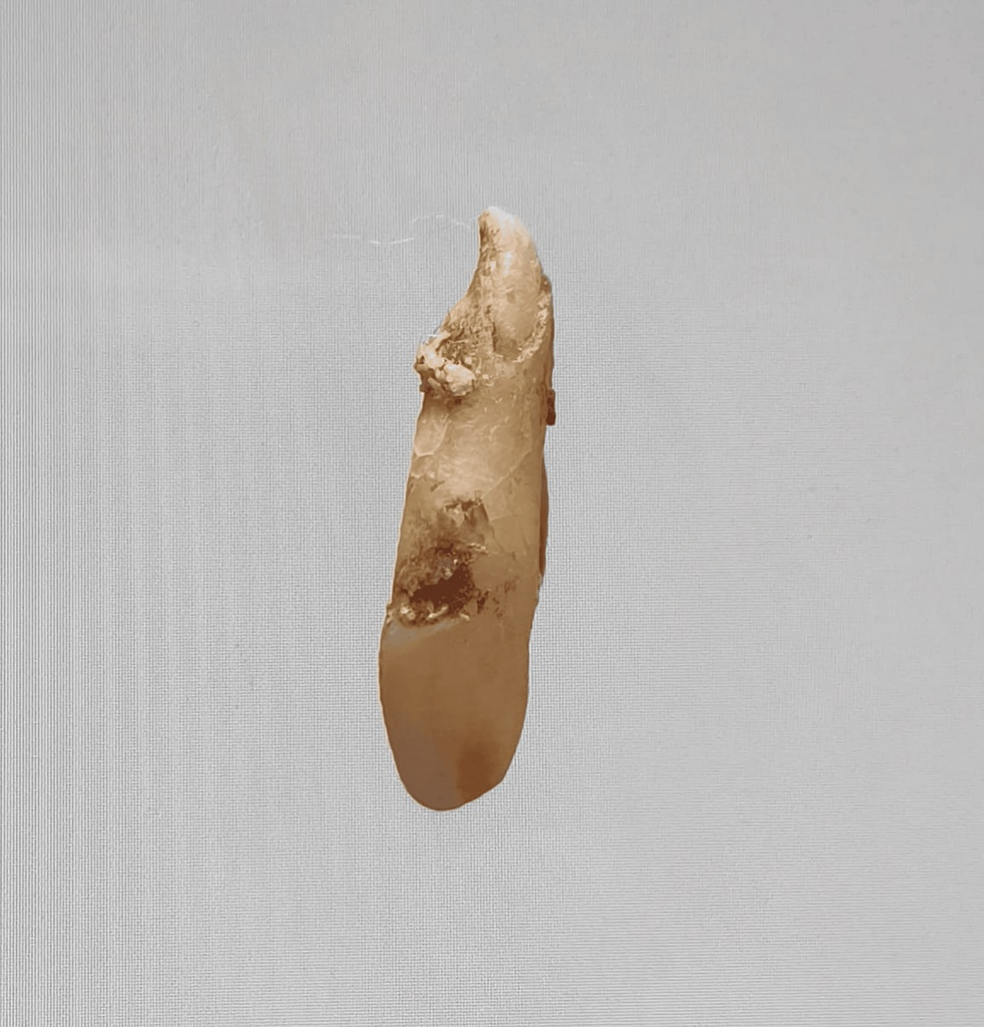
Image showing peg lateral.

**Figure 4:**
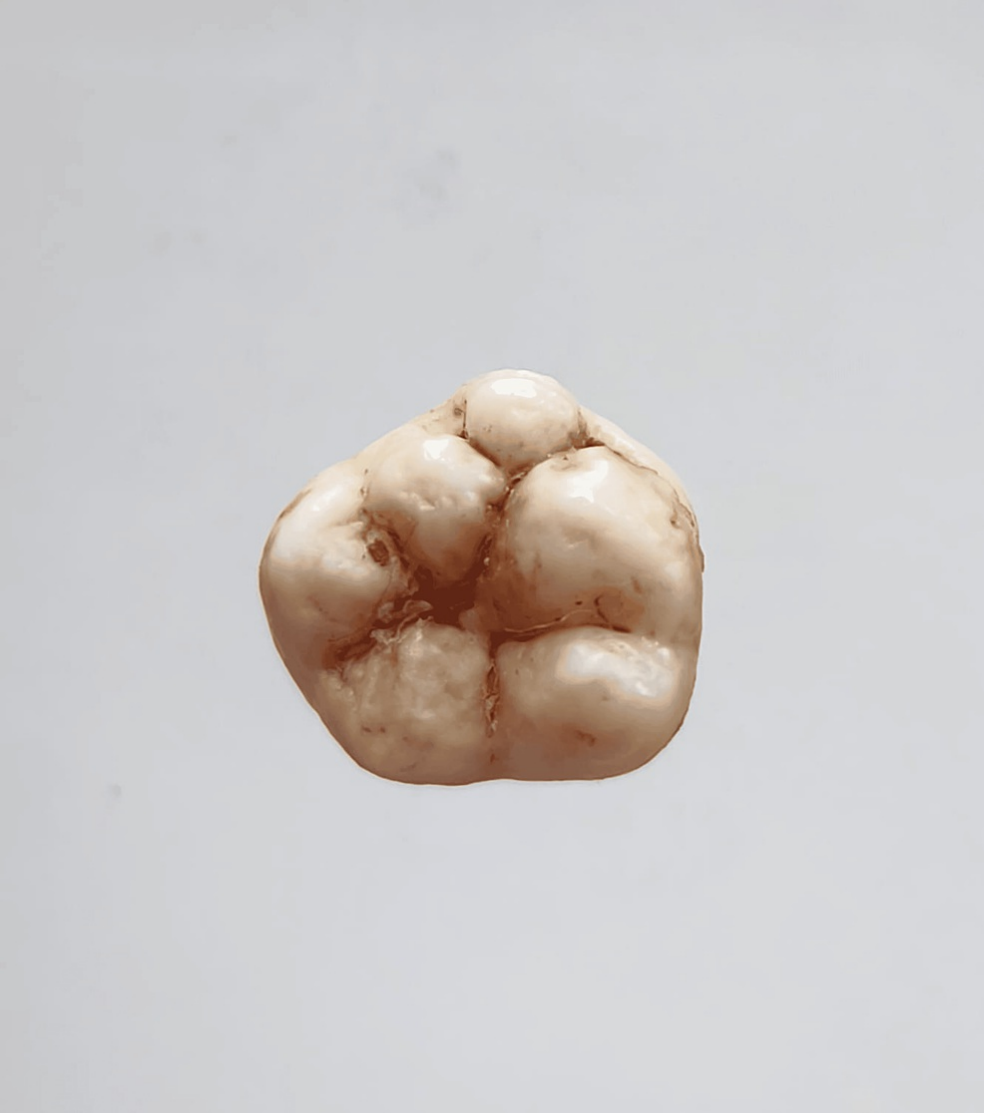
Image showing parastyle.

**Figure 5:**
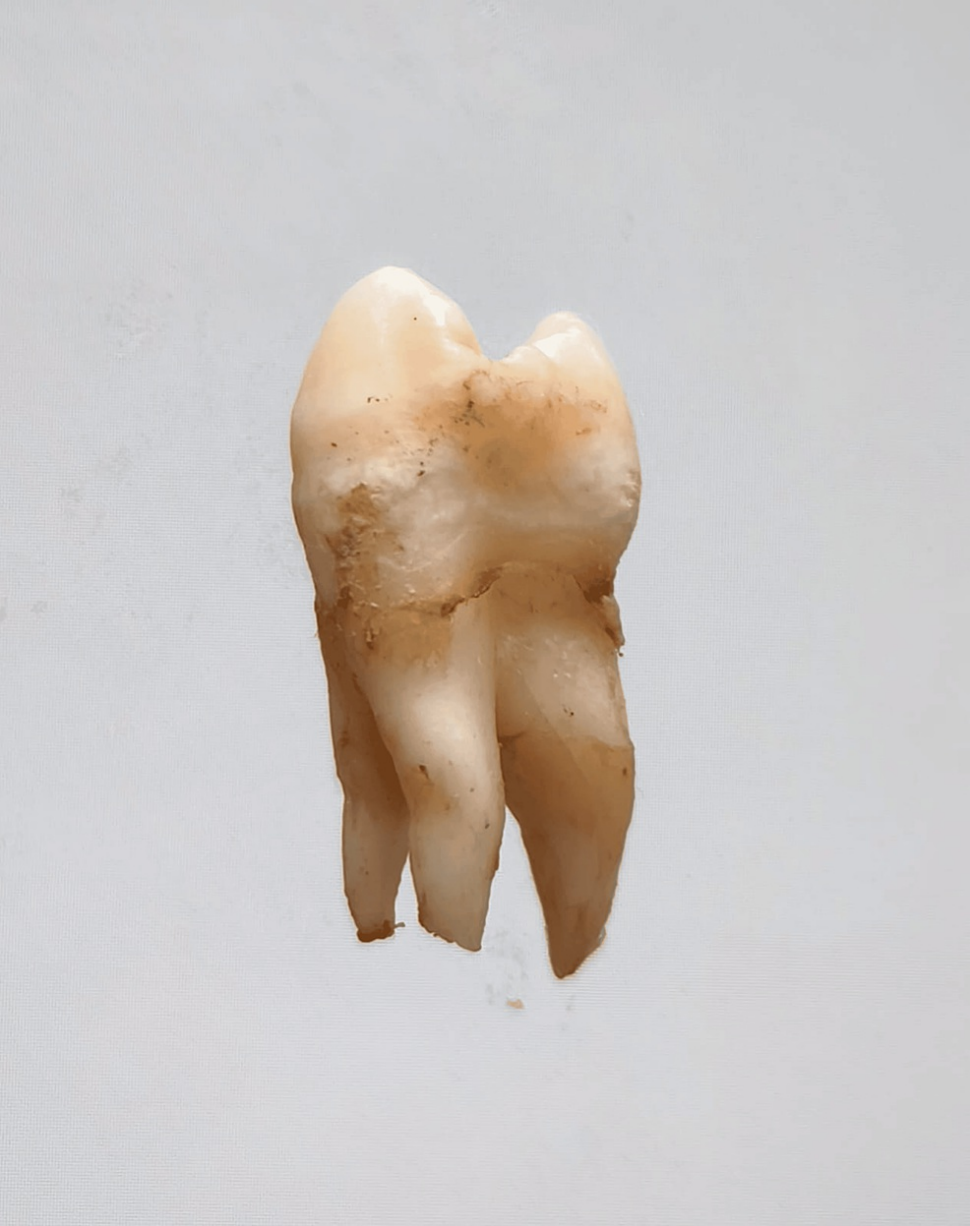
Image showing radiculous premolar.

**Figure 6:**
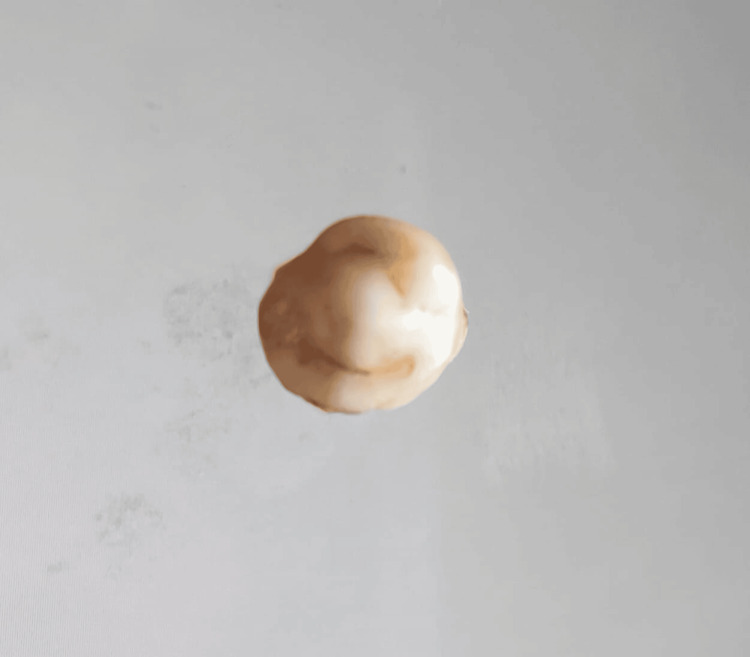
Image showing dens evaginatus.

**Figure 7:**
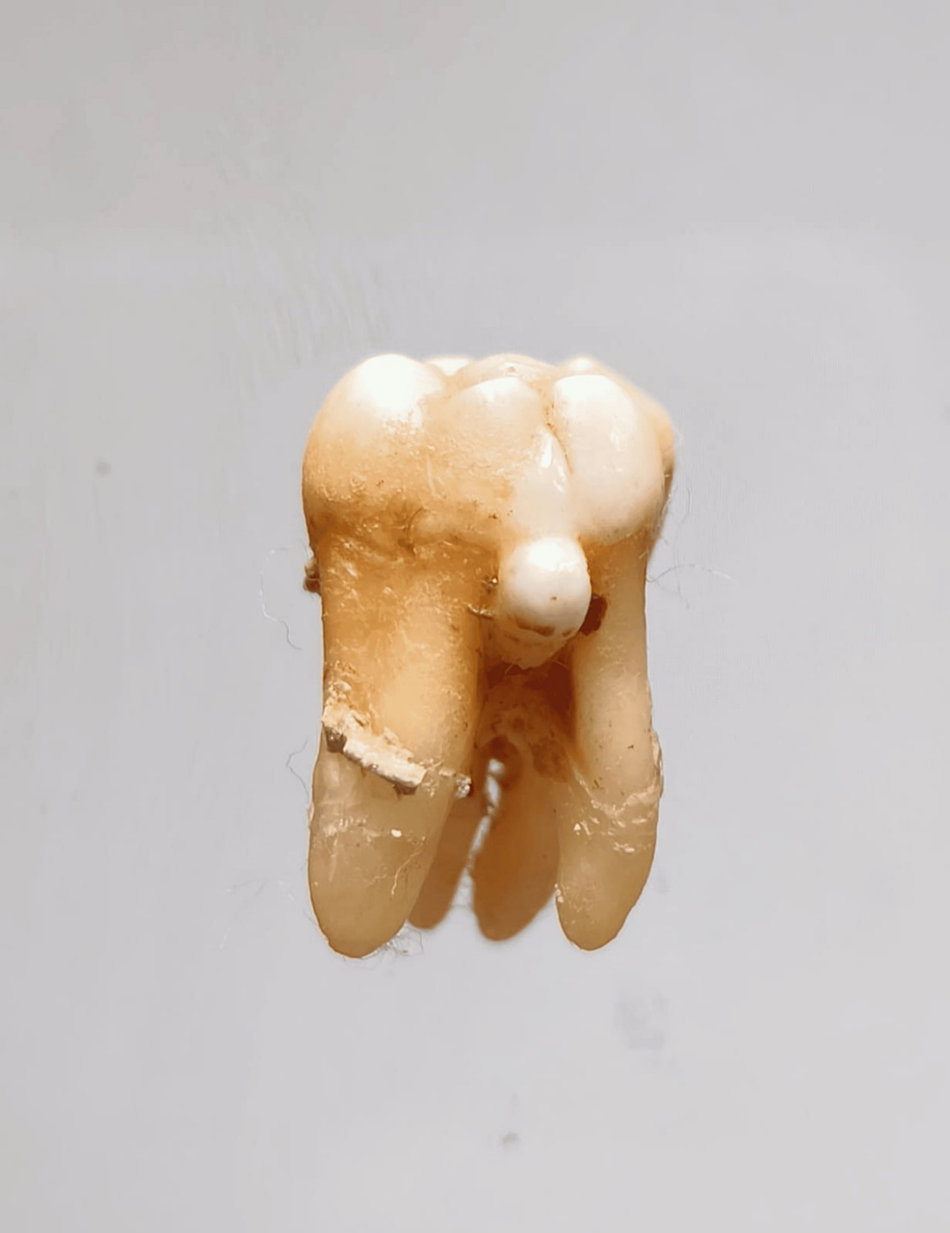
Image showing enamel pearl.

**Figure 8:**
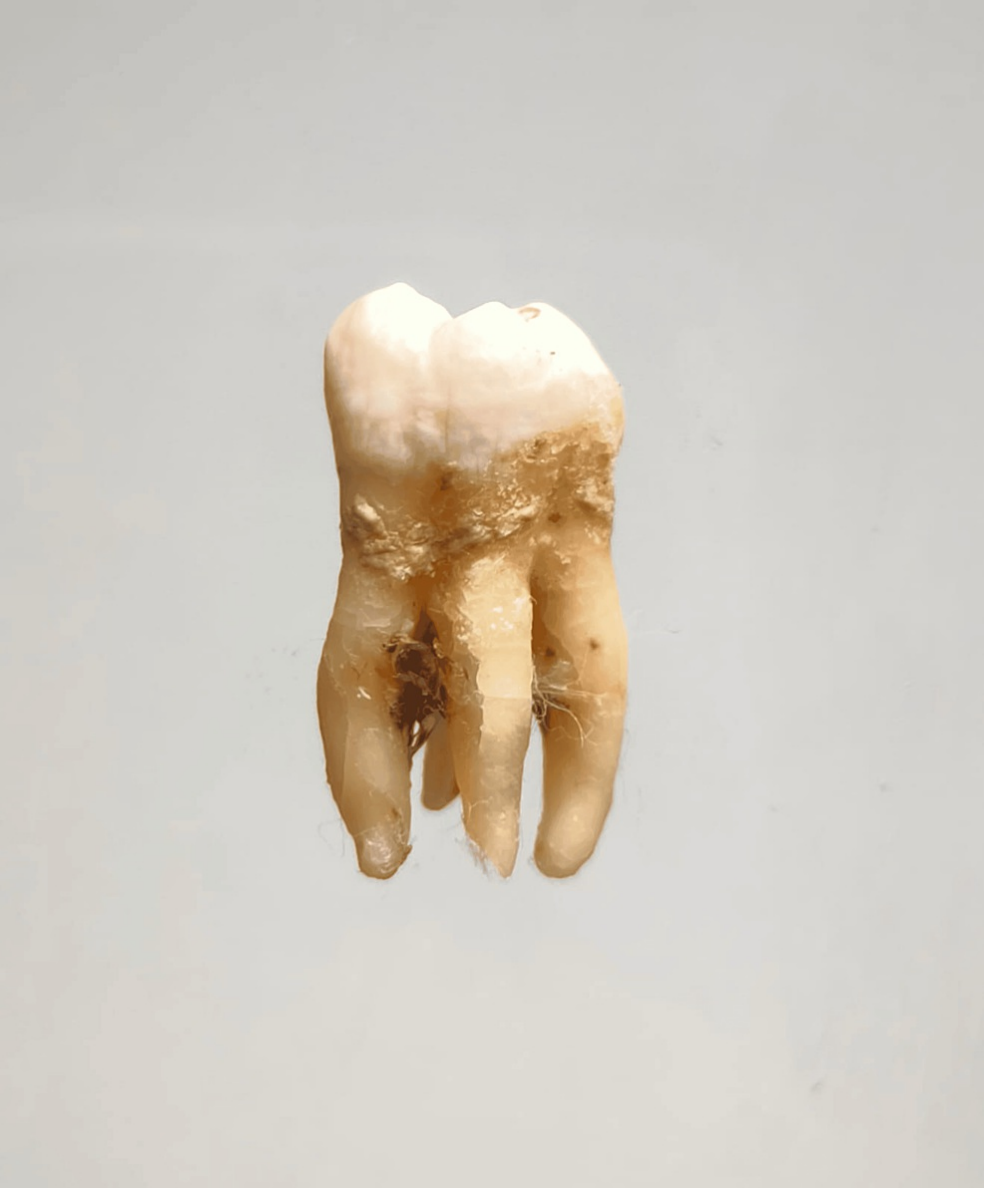
Image showing radix entomolaris.

**Figure 9:**
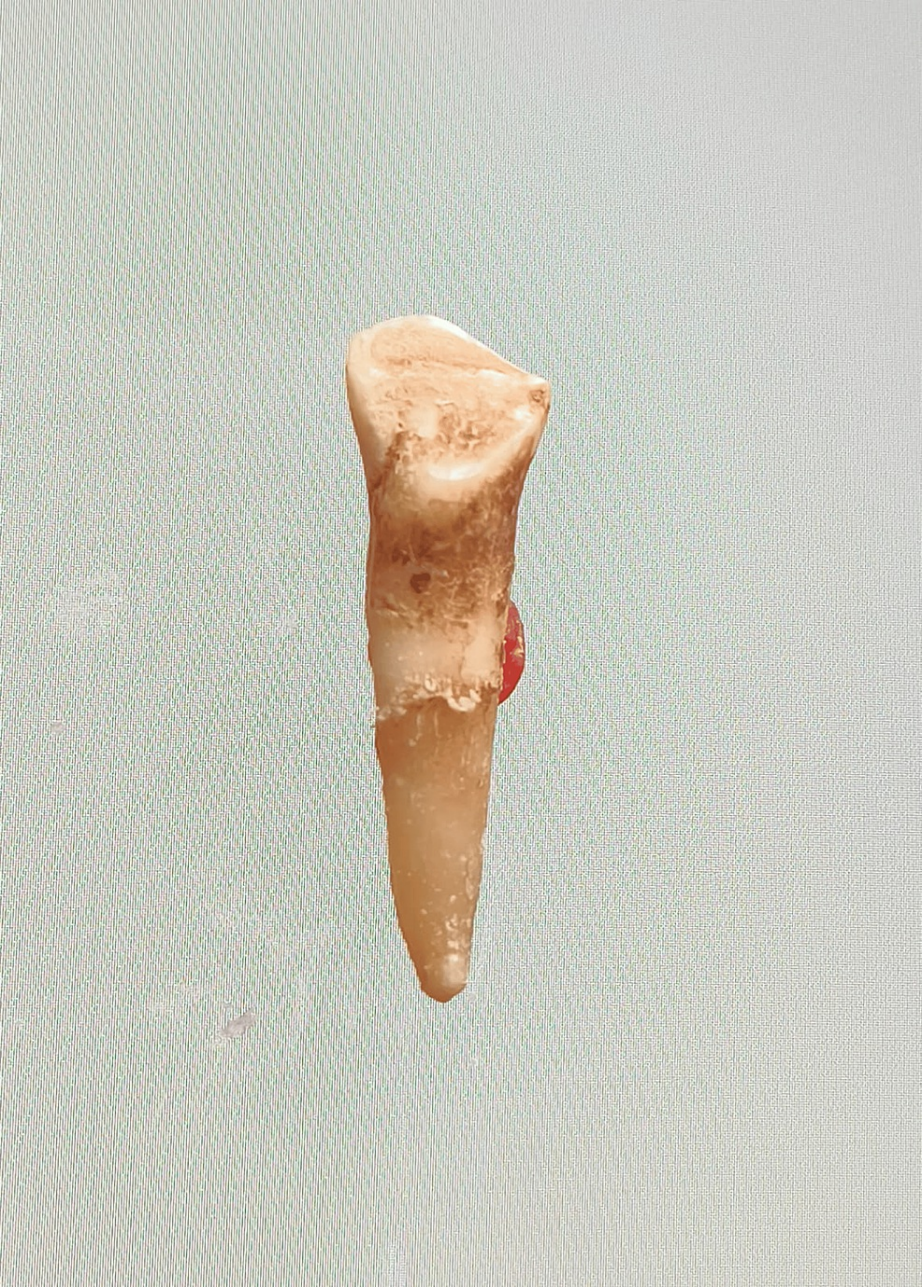
Image showing Bushman's canine.

**Figure 10:**
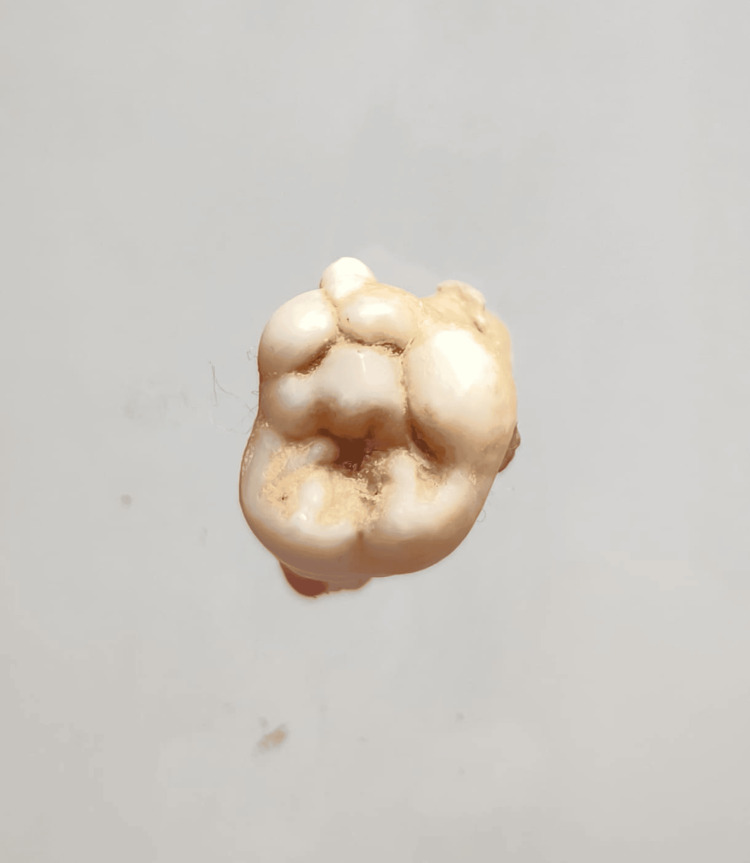
Image showing multiple parastyle.

**Figure 11:**
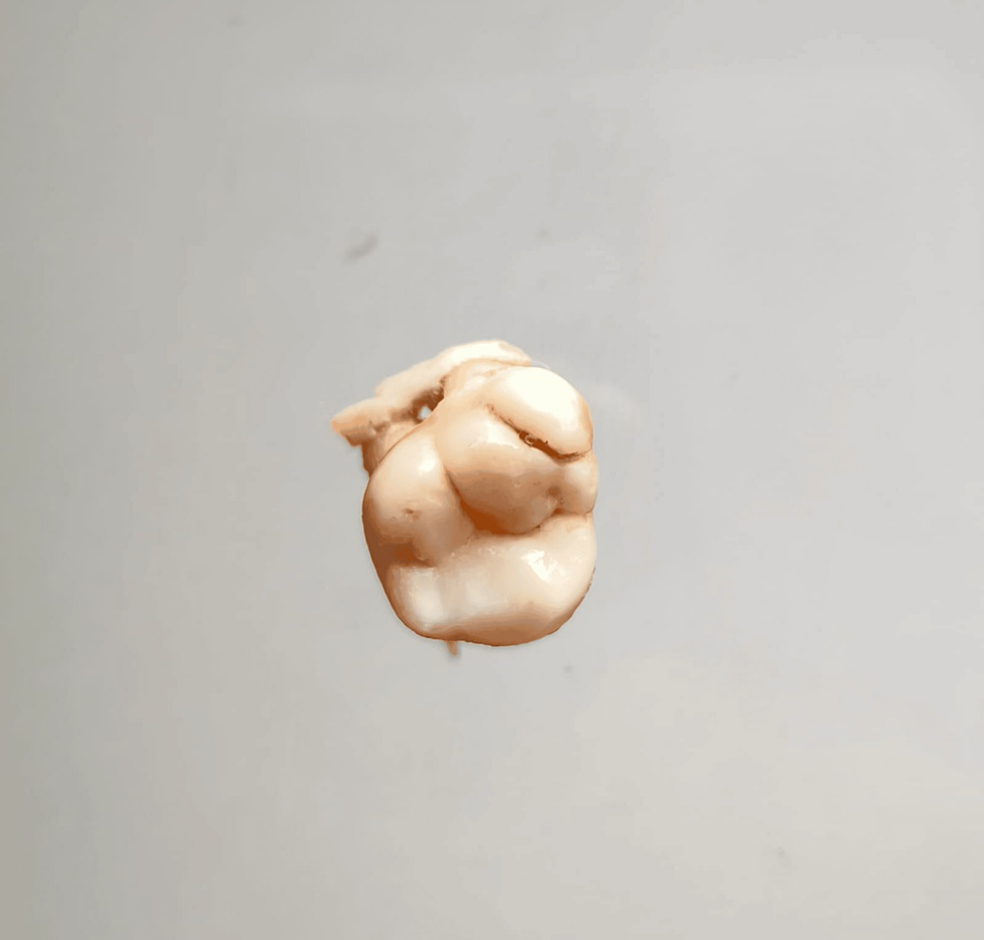
Image showing tuberculum intermedium.

**Figure 12:**
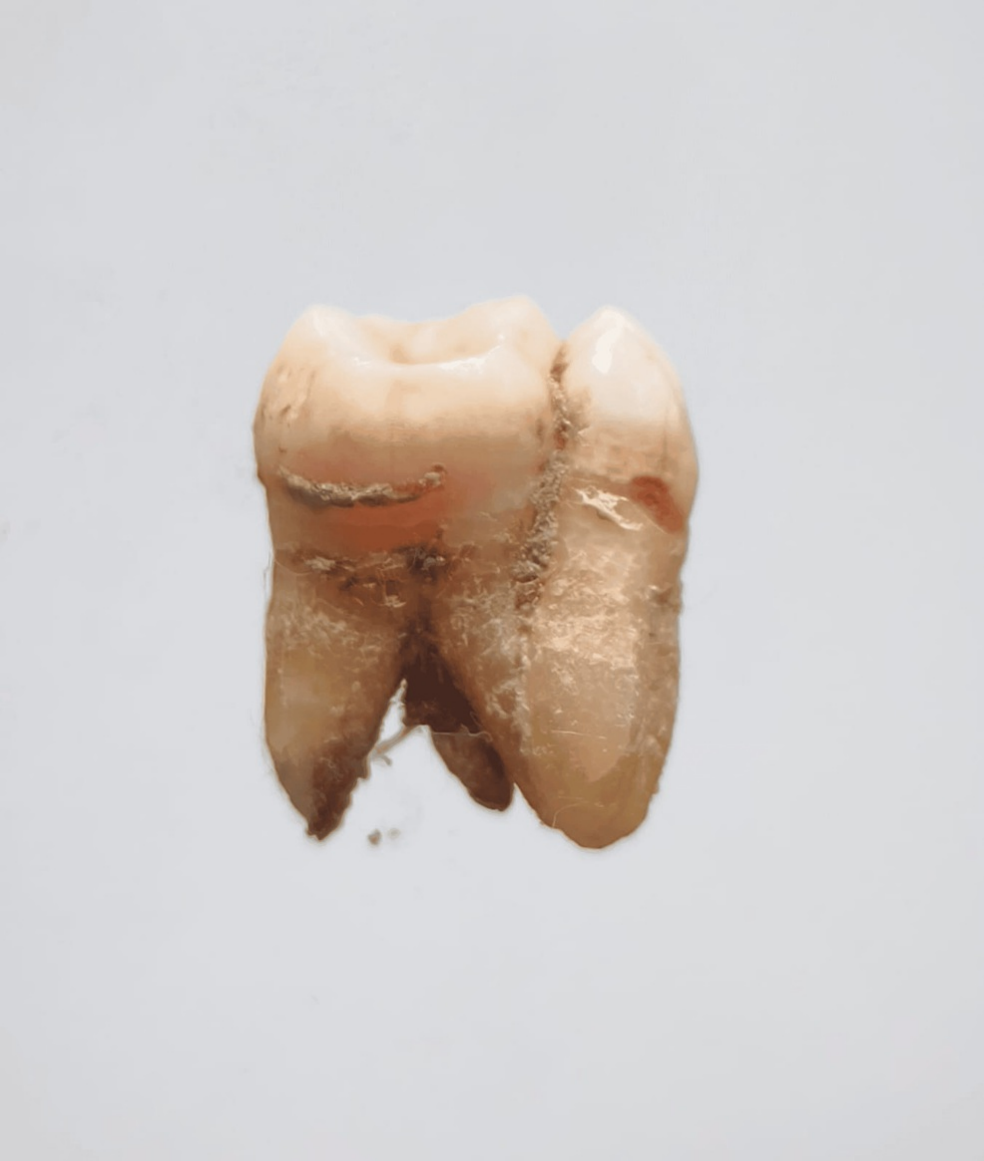
Image showing fusion.

**Figure 13:**
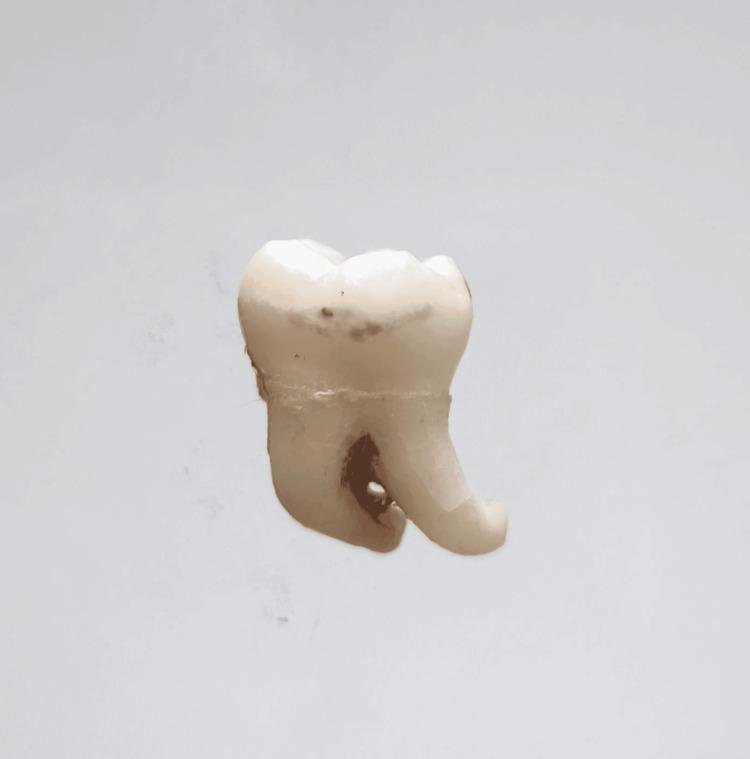
Image showing dilaceration.

The results of the current study are discussed in Table 3.

**Table 3:** Frequency of occurrence of non-morphic dental traits.

Traits observed	Number of teeth in which traits observed
Cusp of Carabelli (CC)	260 (52%)
Talon's cusp (TC)	0
Shoveled incisor (SI)	41 (8.2%)
Peg-shaped lateral incisor (PL)	37 (7.4%)
Protostylid (PR)	0
*Dryopithecus* groove pattern (DP)	0
Hypoconulid (H)	0
Parastyle (PA)	4 (0.8%)
Multiple parastyle (MPA)	1 (0.2%)
Paracone (PC)	0
Bushman's canine (BC)	2 (0.4%)
Interruption groove (IG)	11 (2.2%)
Tuberculum dentale (TD)	0
Radix entomolaris (RE)	198 (39.6%)
Fusion (F)	14 (2.8%)
Tuberculum intermedium (TI)	3 (0.6%)
Radiculous premolar (RP)	1 (0.2%)
Dilaceration (D)	291 (58.2%)
Dens evaginatus (DE)	6 (1.2%)
Enamel pearl (EP)	4 (0.8%)

## Discussion

The idea that dental morphology is heritable may help reconstruct historical migration patterns and have a major impact on our comprehension of human evolution [14]. The morphological characteristics of teeth do not change throughout time without a reason. Many ideas explain why different races exhibit traits differently; among them are disagreement in the literature regarding the extent of genetic control in the manifestation and appearance of these characteristics [15]. Many researchers used visual examination to analyze non-metric features, but few analyzed from dental casts. Some studies combined the two methods. Hypotheses have been proposed as an explanation for these variations among various race relations. According to the clonal model theory, because the characteristic is innate, it is less sensitive to external elements. Characteristics stem from the interplay between genetic and environmental elements. It has been proposed that a population-level visualization of the trait's frequency is possible [5].

It is additionally guaranteed that gender accuracy and racial history recording cannot be ascertained with any degree of reliability by prior castings [16]. The most notable characteristics were the shoveled incisor, peg-shaped laterals, cusp of Carabelli, radix entomolaris, and dilaceration. In a research on the non-metric characteristics of posterior teeth among the Kerala population [5], 17.78% of the population who showed the Carabelli trait also presented with *Dryopithecus* groove in the letter "y" pattern. In a study, 40.5% of the Carabelli trait and 68.2% shoveling of incisors were discovered locally in Bangalore, Karnataka [1]. Around 49.7% of the population who showed shovel and Carabelli features belonged to the Chinese population. The fifth cusp (Carabelli cusp) prevalence in the maxillary molars in 41.7% of school children in Saudi Arabia was observed [17]. According to the well-known paleontologist William King Gregory (1922), there was not much variation in tooth crown shape between the various human groups [5].

Similar to Angadi and Acharya (2008), who reported that 34% of upper second molars in their previously stated Indian sample (n=100) displayed hypocone absence, three-cusp upper second molars were found in 32.8% of the Ajnala teeth. Once more, compared to reports for Western Eurasians (17%), sub-Saharan Africans (6.7%), and North-eastern Asians (12.7%), this frequency is distinct and greater [2].

The strongest and most mineralized tissues in the human body are those that comprise the dentition. Teeth make up approximately 90% of the fossil record and can hold their shape for extended periods. Thus, anthropologists place a great deal of importance on the morphology of the human mouth. Anthropologists utilize the Arizona State University Dental Anthropology System (ASUDAS) to gather information about human dentition through morphological grading. The characteristics recorded by the ASUDAS are observable and trustworthy. They can be distinguished if they lack sexual dimorphism and have deteriorated teeth. These characteristics also strongly characterize populations for affinity research [1].

Non-metric features are mainly employed to predict human identity, gender, and origin, while some non-metric traits may have sex dimorphism, as this study has shown [10]. Gender differences appear to be infrequent in the non-metric features of dental crowns. There is not much of a statistical correlation between these qualities. The frequency of these traits varies significantly with location. These traits are also very important for racial and legal identity [10].

The Carabelli trait was initially noted by Rousseau in 1827; however, Georg Carabelli, the Austrian Emperor Franz's dentist, made the observations that are known as Carabelli's trait or cusp in 1842. It's still unclear how Carabelli's characteristic works. The authors postulate the following: the trait is primitive and molar reduction is indeed the cause of its disappearance; it evolved lately to compensate for the secular trend of dental size decrease; and Carabelli's trait can provide stronger biomechanical stress resistance for the first upper molar. Asian people were found to differ from European ones because of their low frequency of the Carabelli trait and high shoveling presence [18].

According to a study, compared to Tibeto-Nepalese, who had a cusp of Carabelli on 37.19% of their population, 56.86% of Indo-Nepalese had this condition based on ethnicity. Likewise, shoveling in the upper incisors was observed in 30.71% of Indo-Nepalese people and 65.28% of Tibeto-Nepalese people. Thus, this study shows that the Indo-Nepalese population has a large percentage of Carabelli's cusp, while the Tibeto-Nepalese population has a higher prevalence of shoveling [8].

In an Australian population study, primary molars expressed the Carabelli characteristic more than permanent molars [19]. Kieser noted that the Carabelli trait was highly uniform in its appearance throughout the primary and permanent dentition, based on his observations of the trait's expression on molars. This conclusion, he surmised, was compatible with a high genetic influence on Carabelli characteristic expression and a low epigenetic one [20]. The conspicuous characteristic of shoveled incisors and canines varies geographically and seems constant within a community. Shoveling is a common characteristic among Asians and Americans, according to earlier research [21]. A study conducted on the population of Saudi Arabia demonstrates that the variations in the phenotypic appearance of the trait in different dentition types and sexes, as reported by certain workers, may indicate that the trait is subject to a polygenic impact [22]. According to studies by Kharat et al., between 20% and 25% of Sudanese and Egyptians showed shoveling frequency [23]. The ethnic South Indian population in the current study showed shoveled incisors 8.2% of the time. Therefore, compared to Mongoloids and Caucasian Americans, the South Indian ethnic population has reported a reduced commonality of shoveling. According to our study, 2.2% of the population has an interruption groove. According to Guo et al.'s Chinese study, the occlusal morphology of the *Dryopithecus* groove was the least characteristic. Microdontia is most frequently linked to laterals with a peg form. This kind of tooth morphology might result from a developmental disruption that occurs during odontogenesis, primarily damage to the growing tooth [20]. Peg laterals likewise demonstrated a typical gender predilection, with females showing a higher (6%) preference than males in line with earlier research. Thus, we deduce that gender, race, and ethnicity all affect peg laterals differently.

Limitations of the study include subjectivity in trait identification since different observers may perceive traits differently, which might affect the consistency of the results. The variations between the right and left quadrants of the same arch have not been studied. Further, non-metric features frequently have unclear classification criteria, which makes their use unclear. Constraints on sample size may impede statistical analyses and reduce their capacity to be generalized. Results can be skewed by preservation biases in archaeological materials, which tend to reflect traits unique to a community rather than general trends. Additionally, because environmental variables, such as dietary habits, stress, cultural practices (betel nut chewing), etc., can affect tooth morphology, non-metric features could not adequately represent genetic diversity. These restrictions highlight the necessity of supplementary methods and cautious interpretation in dental anthropological research.

## Conclusions

The present study concludes that three non-morphic dental traits, namely, the cusp of Carabelli, radix entomolaris, and dilaceration, are common in the population observed. There was a moderate to significant positive correlation between the incidence of a few dental traits/anomalies. This data indicates that the majority of the ethnic South Indian population possesses these characteristics, suggesting that they could serve as a useful tool for human identification. This study also exhibits the importance of maintaining antemortem dental records, such that it can help us in human identification of mass fatality, road traffic accidents, and catastrophes.
